# Dr. Allen’s Views upon Homœopathic Practice

**Published:** 1887-11

**Authors:** T. F. Allen


### DR. ALLEN'S VIEWS UPON HOMŒOPATHIC PRACTICE.

To The Homœopathic Physician:

In your issue of September, 1887, just received, you ask of me information as to my beliefs, courteously: To such a request I am happy to reply.

The New York *Medical Times* has incorrectly quoted me in making me say that "I do not believe that Homœopathy is the only law of healing." *I never gave utterance to any such sentiment,* nor do I think it. What I may have said and what I am forced to believe is, that Homœopathy is not the only way to treat the sick. I know of no other *law of cure* than the law of similars; it is a law I stand by in my public utterances and in my private practice. No one can more consistently follow its guidance day by day than I do; no one is more intolerant of empiricism than I; no one gives fewer doses, and, I may almost say, less medicine. But while I (the pronoun "I" is used with the intention to give expression simply to a personal opinion) practice Homœopathy pure and simple for the cure of the sick, avoiding even a highest potency of an *unproved* drug for the "cure" of neuralgia or anything else, other methods of treating the sick are forced upon me. These methods are:

*First. Palliation.* This I put first because by me it is most infrequently used—*once* only during the past two years have I been forced to resort to hypodermic injections of Morphia in the closing hours of a woman suffering from cancer of the stomach. It is indeed rare that a remedy homœopathic to the symptoms fails to relieve even in an incurable case, but it sometimes happens, and when it does happen, whether from the ignorance of the physician or the peculiarity of the case, one's duty is perfectly clear and must be followed. I heard a homœopathic physician once say that if the remedy failed to relieve even in the highest potency, the patient must "howl;" he would not be guilty of administering Opium. I wish clearly to be understood—palliation is seldom necessary; the true homœopathist rarely uses it; but it is one way in which the sick must occasionally be treated.

*Second. The removal of the cause of disease.* Under this heading lies a mine of investigation, which has but just been opened. If we, as homœopathists, ignore the strides of medical progress in the direction of sanitary science and fail to keep abreast of that progress, we shall go under. What we have to do is to demonstrate the truth of God's law in Therapeutics, not to cover it up with dust. The truth is, people will not get sick if they are cured of all their chronic maladies and live properly; the truth is, Homœopathy alone is able to cure these chronic diseases; but it is not the truth that Homœopathy will remove the cause of disease. There are numerous instances in which urgent symptoms, the direct effect of an exciting and persistently acting cause, must be relieved by a forcible removal of the cause, and then the patient treated homœopathically to cure him and prevent a recurrence of the malady. These causes are not limited to gross substances, such as copper cents or green apples, but include substances which are microscopic in size but infernal in activity. Time would fail me now to enter into the whole discussion of contagion and zymosis, but there is no doubt in my mind that while, as a rule, patients are best treated by allowing the morbid process to work itself out, relieving the symptoms homœopathically as they arise day by day, and putting the system into the best possible condition to withstand the attack, there are cases in which, not being able to cure in a few days, the whole cachexia of the poor sufferer, not being able by a dose of Sulphur or any other antipsoric to remove his inherited psora that renders him susceptible to disease and feeble to resist it, we must suppress for a time the violence of the zymosis.

A most worthy but bigoted homœopathic physician allowed a young lady in this city to shake actually for over six months with tertian and double-tertian fever. The patient has never recovered from the absolute anæmia which resulted; more than ten years have not been sufficient to restore her health. The family let him see it through, and she never took any medicine but in the highest potency. Now, in such a case my duty is clear: Stop the overpowering activity of the zymosis, and then cure the patient. Once or twice only in many years have I been obliged to use Quinine in large doses. I cure almost every case within a week or two by the clearly indicated remedy; sometimes they recover in forty-eight hours; but now and then one must do differently.

He who in these days will not wash out with distilled water and one five-thousandth of a grain of corrosive Mercury a fresh case of gonorrhœa and cure his erring brother in twenty-four to forty-eight hours, must give up the treatment of such diseases. The fact that one in a hundred gets an orchitis or rheumatism shows rather that the water was too cold than that the cleaning out of a poison which is unclean and purely local caused a suppression of sycosis or any other -osis. But we do not know much about these poisons which get into us and ferment in our blood and tissues when we are below par. What I think is that, unless we know definitely all about it the best plan is to treat our patients carefully and homœopathically, but when we do know something about it, expel the intruder first.

*Third. Local applications.* It is a pretty theory, and one which works well *as a rule*, that if a patient be placed in a favorable condition all sores will heal and all discharges will be natural. I practice on that plan, and I use local applications very seldom, but I am bound to recognize the fact that *some* patients will absolutely not get well without cleaning the sores and washing off morbid secretions. Doctors may theorize and fuss about this all they please, the fact remains: local applications are necessary in some cases. Always in conjunction with such cleansing applications I prescribe the indicated remedy.

*Fourth. Electricity.* But as I do not believe much in it nor in any of the other expedients I have mentioned, I will not prolong this letter.

If an unbiased observer will watch the practice of different physicians he will see that each gathers about him his own set. Each will say that his world is complete and his methods satisfactory. I witness an eminent physician giving only high potencies gather about him a coterie composed exclusively of those susceptible to such doses and flattering the doctor into the belief of his own infallibility. I witness also the hundreds of people who have failed to receive benefit at his hands quietly dropping away from him, and drifting into the circle of another who, in his turn, has his own following, and who conceives his methods to be nearest perfection.

I believe that as the years go by and good, honest work is done by us, we shall have less need of adjuvants and expedients. I know that within the compass of my own experience families have become less and less liable to sickness, and the children have grown more healthy and beget healthier offspring. Till the medical millennium, however, we must take sickness as we find it, and do our best to cure, and to relieve if we cannot cure.

It will give me pleasure to defend my opinions against all comers who fight fair, but against those who use offensive epithets and impute unworthy motives I have no weapons.

T. F. Allen.

